# Novel Insights into Pathophysiology of Orbital Inflammatory Diseases and Progression to Orbital Lymphoma by Pathway Enrichment Analysis

**DOI:** 10.3390/life12101660

**Published:** 2022-10-20

**Authors:** Karim Al-Ghazzawi, Fabian D. Mairinger, Roman Pförtner, Mareike Horstmann, Nikolaos Bechrakis, Christopher Mohr, Anja Eckstein, Michael Oeverhaus

**Affiliations:** 1 Department of Ophthalmology, University Hospital Essen, 45147 Essen, Germany; 2 Institute of Pathology, University Hospital Essen, University of Duisburg-Essen, 45147 Essen, Germany; 3 Department of Oral and Maxillofacial Surgery, University of Duisburg-Essen, Kliniken-Essen-Mitte, 45136 Essen, Germany

**Keywords:** NSOI 1, IgG4-ROD 2, MALT 3, OID 4, TAO 5, adipocytokine 6, neurotrophin, 7, ECM receptor 8

## Abstract

Non-specific orbital inflammation (NSOI) and IgG4-related orbital disease (IgG4-ROD) are currently treated with non-specific immunosuppressive agents based on non-randomized, uncontrolled studies. Therefore, relapses and prolongated courses are common and remain challenging. For a more specific therapy, a better understanding of the underlying pathophysiology is crucial. Therefore, we aimed to analyze signaling pathways to expand the knowledge on the pathophysiology and possibly identify specific targets in the future, as occurred recently in Graves’ orbitopathy with the IGF-1 receptor. Furthermore, we analyzed potential mechanisms for the described potential progression to orbital MALT (mucosa-associated lymphoid tissue) lymphoma. The investigation cohort for this screening study comprised of 12 patients with either typical NSOI (n = 6), IgG4-ROD or MALT lymphoma (n = 3 each). Mean age was 56.4 ± 17 years. MALT samples, in contrast with IgG4-ROD and NSOI, showed overall upregulation for extracellular matrix receptor interaction (ECM) and adipocytokine signaling. Investigating signaling compounds for MALT samples, differentially expressed genes were re-identified as targets with relevant expression. Even though pathway analysis showed differentially altered products when comparing IgG4-ROD with MALT, main conductors of differentiation in B- and T-cell signaling were commonly altered when observing the microenvironment of examined tissues. Our data reveal the characteristic differences and similarities in genetic-expression-based pathway profiles between MALT lymphoma, IgG4-ROD and NSOI, which may be useful for elucidating the associated pathogenic mechanisms and developing specific treatments for these orbital diseases.

## 1. Introduction

The term orbital inflammatory disease (OID) encompasses a wide range of different orbital diseases. The most common of these is the recently discovered Graves’ orbitopathy (GO), which is probably why our understanding of its pathophysiology has highly advanced in recent years [1,2,3,4,5,6,7]. A key finding was the “crosstalk” of insulin-like growth factor-1 receptor (IGF-1R) and thyroid-stimulating hormone receptor (TSHR), which are both expressed on orbital fibroblasts and are upregulated in active GO patients. They seem to synergistically regulate downstream signaling pathways (including MAPK/Ras/Raf/MEK/ERK and PI3K/Akt/mTOR pathways), leading to increased hyaluronic acid synthesis, inflammation and adipogenesis [3,4]. This new insight lead to clinical trials of a fully human monoclonal IGF-1R antibody, teprotumumab, as a targeted therapy, which has already been approved by the FDA in the U.S. but not by the European Medical Agency (EMA) [8,9,10]. In contrast, non-specific orbital inflammation (NSOI, or idiopathic orbital inflammation (IOI); former pseudotumor orbitae) and IgG4-related orbital disease (IgG4-ROD) are much less common and less understood [11]. Diagnosis and therapy remain challenging [12,13]. Although recent studies have advanced our knowledge of the underlying pathophysiology, IOI remains an exclusion diagnosis and diagnosis of IgG4-ROD needs an orbital biopsy in most cases [14,15,16,17,18,19,20]. Clinical presentations and histological findings for NSOI are heterogeneous, and we are still lacking specific diagnostic factors. Therefore, orbital mass lesions are sometime misinterpreted patients receiving inadequate therapy for a certain amount of time before being re-evaluated correctly. Patients suffer similar to IgG4-ROD patients from proptosis, pain, diplopia and vision loss depending on the stage and location of the diseases. The lack of specific targeted therapies is the reason these entities are treated with broad immunosuppressive agents, and why patients suffer from relapses and inadequate treatment responses. Therefore, we aimed to research the underlying pathophysiology on a molecular level by genetic expression pathway analysis to identify shared and different pathways. This could lead to the identification of targets as in GO for specific therapies in the future. Due to the rareness of these entities, we used NanoString nCounter technology, which allows high-throughput, precise and reliable RNA analysis even of formalin-fixed, paraffin-embedded tissue (FFPE) [21]. This allowed us to use routinely acquired and stored orbital biopsies, which is a great advantage for the elucidation of such a rare disease. The technology detects abnormally altered genes or molecular pathways, and is therefore an ideal tool for the gene expression and transcriptome analysis of these orbital diseases. As a secondary objective, we aimed to gain more knowledge about the oncogenesis progression of IgG4-ROD to orbital MALT lymphoma (mucosa-associated lymphoid tissue), which remains a partially understood phenomenon despite all recent advances [22,23]. Current hypothesis stipulates that MALT lymphoma arises from chronic inflammation as in IgG4-ROD and malignant transformation is mediated by acquired mutations (i.e., activation of nuclear factor-kB (NF-kB) pathways) [24]. These could be caused by viral or bacterial infection (i.e., HTLV1 and EBV [25,26]), which directly infect lymphocytes, inducing lymphoid hyperplasia and malignant transformation over time [27].

## 2. Materials and Methods

### 2.1. Study Population

This study is based on our previously published study [28]. In the previous study, we identified 12 patients (mean age 56.4 ± 17 years) with typical clinical course and certain diagnosis for NSOI (n = 6), IgG4-ROD and MALT lymphoma patients (n = 3 each) from our patient database comprised of patient records between 2000–2020. Please refer to [28] for more details on the clinical characteristics. The study was performed under adherence of the ethical foundations of the Declaration of Helsinki and was approved by the Ethics Commission of the University of Essen (11-4822-B0). Diagnosis of NSOI, IgG4-ROD and orbital MALT lymphoma were based on clinical, flow cytometric and histological (including immunostaining) examinations. IgG4-ROD was diagnosed in accordance with the published criteria [18]. Briefly, IgG4-ROD was diagnosed in the presence of (1) enlargement of orbital tissues with marked lymphoplasmatic infiltration and fibrosis/sclerosis, (2) >50 IgG4 positive plasma cells per high-power field (IgG4+/IgG Ratio >40%) and serum IgG4 level >135 mg/dL.

### 2.2. RNA Extraction

RNA extraction of the 12 routinely processed formalin-fixed and paraffin-embedded biopsy specimens was performed as previously described [29]. In short, one to three paraffin sections with a thickness of 7 μm per sample were deparaffinized with xylene prior to RNA extraction using the RNeasy FFPE kit (Qiagen, Hilden, Germany) according to the manufacturer’s recommendations with slight adjustments. Total RNA concentrations were measured using a Nanodrop 1000 instrument (Thermo Fisher Scientific, Waltham, MA, USA) [30].

### 2.3. Digital Gene Expression Analysis

Based on current literature, we selected multiple genes involved in tumor- and inflammation-associated pathways. Gene expression patterns were screened using the NanoString nCounter platform for digital gene expression analysis with the appurtenant PanCancer Progression Profiling panel, consisting of 770 genes and the Immunology V2 Profiling panel consisting of 594 genes mediating immune response as well as 30 reference genes (see Supplementary Table S1). Hybridizations were performed using the high-sensitivity protocol on the nCounter Prep-Station. Post-hybridization processing was performed by using the nCounter MAX/FLEX System (NanoString) and the cartridge was scanned on the Digital Analyzer (NanoString). The cartridge was read with maximum sensitivity (555 FOV). In total, 100 ng sample input was used for each reaction.

### 2.4. NanoString Data Processing

Count data acquired by NanoString analysis were normalized and analyzed using the R statistical programming environment (The R Foundation for Statistical Computing, Institute for Statistics and Mathematics, Vienna, Austria; v. 4.0.3). Considering the counts obtained for positive control probe sets, raw NanoString counts for each gene were subjected to a technical factorial normalization, carried out by subtracting the mean counts plus two-times the standard deviation from the CodeSet inherent negative controls. Subsequently, a biological normalization using the included RNA reference genes was performed. In addition, background noise was excluded by utilization of one-sided Wilks *t*-test of negative controls and target specific counts in all samples to identify genes not relevantly expressed (*p* < 0.05) [31].

### 2.5. Statistical Evaluation

Statistical and graphical analyses were also performed within the R statistical programming environment (v. 4.0.3). Prior to exploratory data analysis, the Shapiro–Wilks test was applied to test for normal distribution of each data set for ordinal and metric variables. Resulting dichotomous variables underwent either the Wilcoxon/Mann–Whitney rank sum test (non-parametric) or two-sided Student’s *t*-test (parametric). For comparison of ordinal variables and factors with more than two groups, either the Kruskal–Wallis test (non-parametric) or ANOVA (parametric) was used to detect group differences. Correlations between metrics were tested applying Spearman’s rank correlation test as well as Pearson’s product-moment correlation testing for linearity. Basic quality control of run data was performed by mean vs. variance plotting in order to find outliers in target or sample level. True differences were calculated by correlation matrices analysis. Quality control of run data was first performed in basic by mean vs. variance plotting to find outliers in target or sample level. True differences and clusters on both target and sample level were calculated by correlation matrices analysis. Pathway analysis is based on the KEGG database (Kyoto Encyclopaedia of Genes and Genomes) and was performed using the “pathview” package in R. Differences were specified by log2-fold changes between means (parametric) or medians (non-parametric) of compared groups. Significant pathway associations were identified by gene set enrichment analysis using the WEB-based GEne SeT AnaLysis Toolkit (WebGestalt) [32,33,34,35]. Each run was executed with 1000 permutations. Finally, all associations were ranked according to the false discovery rate (*p* < 0.05).

Due to the multiple statistical tests the *p*-values were adjusted by using the false discovery rate (FDR). The level of statistical significance was defined as *p* ≤ 0.05 after adjustment.

## 3. Results

### 3.1. Gene Set Enrichment Analysis (GSEA):

For each analyzed group, a gene set enrichment analysis (GSEA) was performed. GSEA utilizes molecular interaction networks outlined by the Kyoto Encyclopedia of Gene and Genomes (KEGG) to map out increased gene expression in a specific molecular pathway, depending on a response variable (entity) [36]. Thereby, we could identify the top activated or downregulated pathways for each entity.

#### 3.1.1. GSEA in Non-Specific Orbital Inflammation

**Figure 1:**
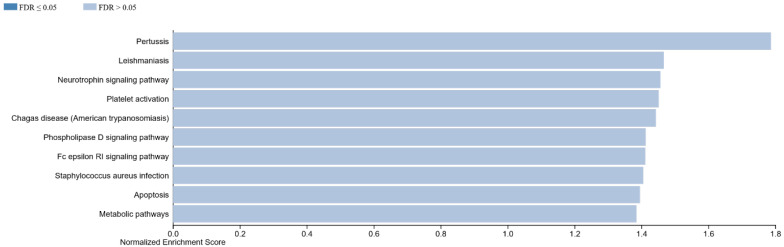
Top enriched pathways for NSOI samples.

For NSOI we noticed an activation of several pathways (Figure 1). These were categorized as follows:

- Innate immune pathways providing general response to foreign bodies:
  pertussis (normalized enrichment score, NES: 1.79; *p*-value: 0.001), Chagas disease (NES: 1.44; *p* = 0.03), Staphylococcus aureus infection (NES: 1.41; *p* = 0.05), phospholipase D signaling pathway (NES: 1.41; *p* = 0.06) and the neurotrophin signaling pathway (NES: 1.46; *p* = 0.04)
- Metabolic pathway part of an effector cascade:
  apoptosis (NES: 1.40; *p* = 0.066), Fc epsilon RI signaling pathway (NES: 1.41; *p* = 0.056), platelet activation (NES: 1.45; *p* = 0.037).

#### 3.1.2. GSEA in MALT Lymphoma

**Figure 2:**
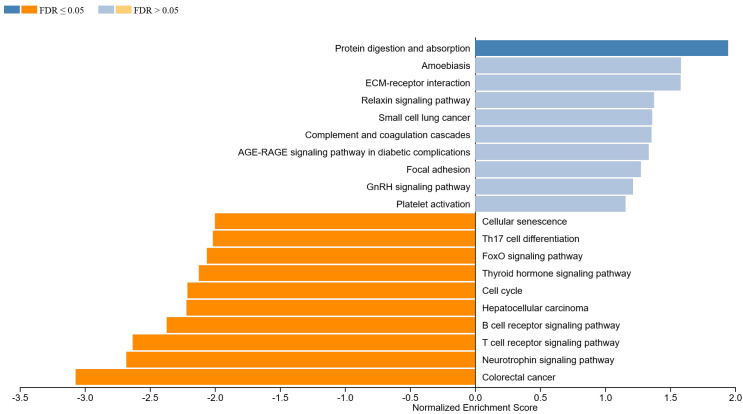
Top enriched pathways for MALT samples.

For lymphoma, top activated pathways were also categorized, respectively, to its attributes (Figure 2):

- Focal surface regulatory pathways: protein digestion and absorption (NES: 1.95; *p* < 0.001), ECM (extracellular matrix)–receptor interaction (NES: 1.58; *p* = 0.002), complement and coagulation cascades (NES: 1.36; *p* = 0.054) and focal adhesion (NES: 1.28; *p* = 0.063), amoebiasis (NES: 1.58; *p* = 0.011)
- Effector pathways with systematic impact: complement and coagulation cascades (NES: 1.36; *p*- = 0.054), AGE-RAGE signaling pathway in diabetic complications (NES: 1.34; *p* = 0.055), small-cell lung cancer (NES: 1.36; *p* = 0.080), relaxin signaling pathway (NES: 1.38; *p* = 0.05)

Top downregulated pathways were noted as the following:

- Focal surface regulatory pathways: B-cell receptor signaling pathway (NES: −2.37; *p* < 0.001), T-cell receptor signaling pathway (NES: −2.63; *p* < 0.001), neurotrophin signaling pathway (NES: −2.68; *p* < 0.001), thyroid hormone signaling pathway (NES: −2.12; *p* < 0.001 and Th17 cell differentiation (NES: −2.02; *p* < 0.001)
- Effector pathways with systematic impact: cell cycle (NES: −2.21; *p* < 0.001), hepatocellular carcinoma (NES: −2.22; *p*-value: <0.001) and FoxO signaling pathway (NES: −2.06; *p* < 0.001)

#### 3.1.3. GSEA in IgG4-ROD

**Figure 3:**
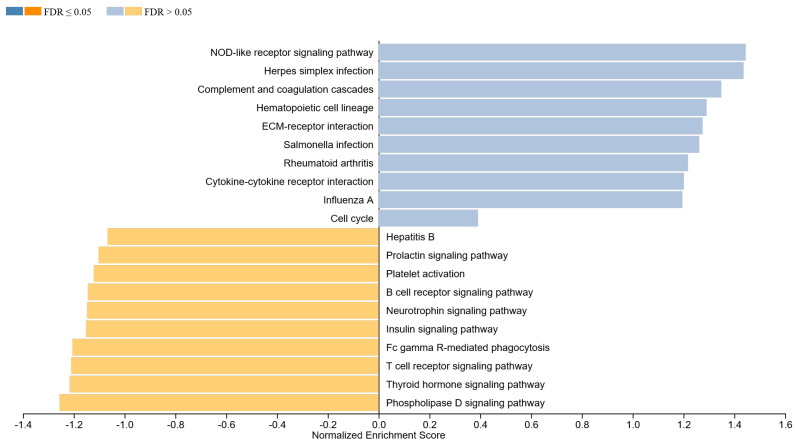
Top enriched pathways for IgG4-ROD samples.

For IgG4-ROD, top eight activated pathways comprise (Figure 3):

- Innate immune pathways providing general focal response to foreign bodies:
- NOD-like receptor signaling pathway (NES: 1.44; *p* = 0.043), complement and coagulation cascades (NES: 1.35; *p* = 0.091), ECM–receptor interaction (NES: 1.28; *p* = 0.126), Salmonella infection (NES: 1.26; *p* = 0.16) as well as cytokine–cytokine receptor interaction (NES: 1.20; *p* = 0.20)
- Effector pathways with systematic impact: hematopoietic cell lineage (NES: 1.29; *p* = 0.129) herpes simplex infection (NES: 1.44; *p* = 0.047), and rheumatoid arthritis (NES: 1.22; *p* = 0.182)

Moreover, top eight downregulated pathways were all noted as focal surface regulatory pathways:

- Fc gamma R-mediated phagocytosis (NES: −1.21; *p* = 0.19), B-cell receptor signaling pathway (NES: −1.14; *p* = 0.193), T-cell receptor signaling pathway (NES: −1.21; *p* = 0.214), neurotrophin signaling pathway (NES: −1.15; *p* = 0.21), platelet activation (NES: −1.12; *p* = 0.34), insulin signaling pathway (NES: −1.15; *p* = 0.350), phospholipase D signaling pathway (NES: −1.26; *p* = 0.111), as well as the thyroid hormone signaling pathway (NES: −1.22; *p* = 0.14)

### 3.2. Re-Identification of Genes with Relevant Expression

**Figure 4:**
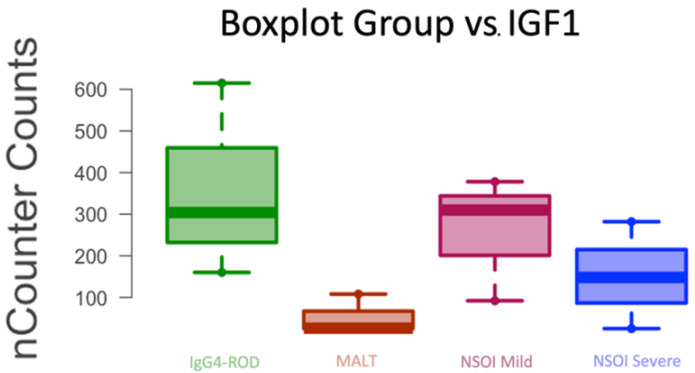
Boxplot of the gene expression for *IGF1* in IgG4-ROD, MALT and NSOI (NSOI samples have been subgrouped into mild and severe clinical courses).

Top differentially expressed genes (FDR *p* < 0.05) were previously reported between each of our groups [28]. To explore the biological functions of these genes, pathway analyses in the form of gene set enrichment analysis (GSEA) was performed as previously described. Relevant pathways were observed and following compounds were re-identified. Focusing on explicit count expression analysis of IGF1 in our study, MALT lymphoma showed significantly lower expression of *IGF1* (*p* < 0.011, FDR *p* < 0.022) when compared to NSOIs and IgG4-ROD. For IgG4-ROD vs. all, expression was not significantly altered (*p* = 0.16, FDR 0.23); looking at our data (Figure 4), we see that this results from elevated expression levels in NSOI samples. Insulin-like growth factor binding proteins (*IGFBP*) were also significantly differentially expressed as *IGFBP4* (*p* < 0.0001, FDR *p* < 0.012) and *IGFBP7* (*p* < 0.005, FDR *p* < 0.02) (Table 1).

#### ECM–Receptor Interaction and Adipocytokine Signaling Pathway

**Figure 5:**
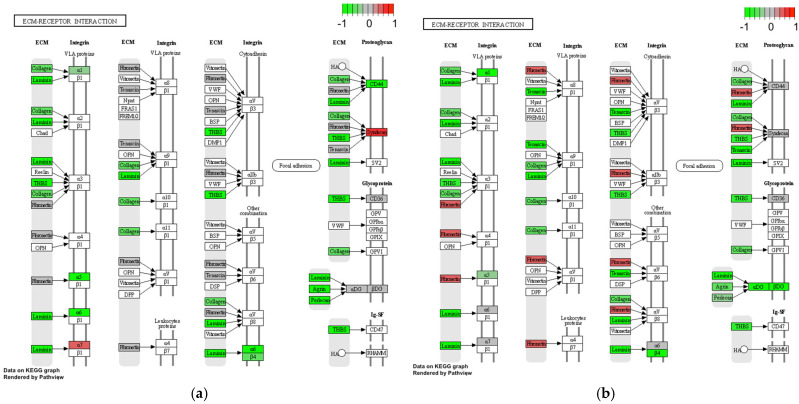

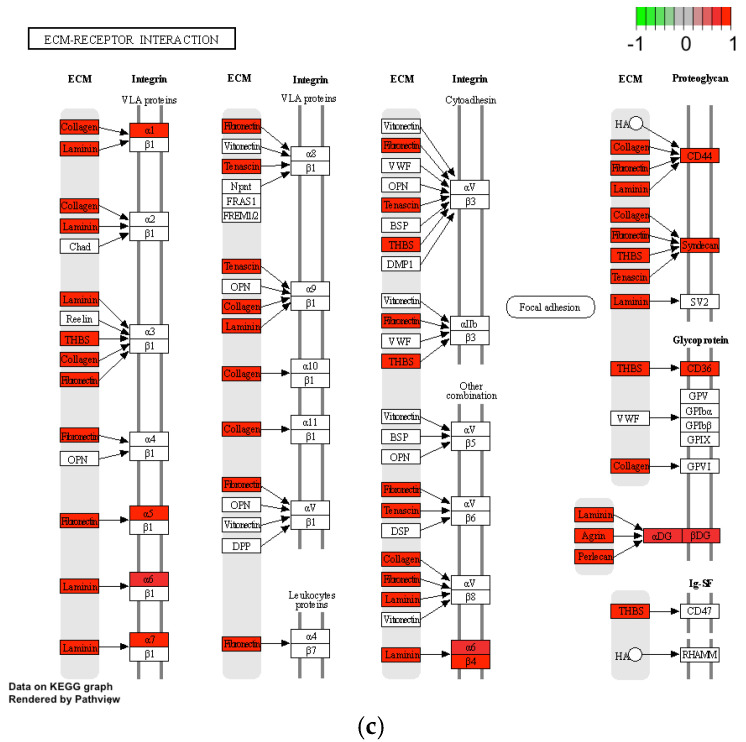
Differential gene expression of genes contributing to the ECM–receptor interaction of different entities. The plots were created via the pathview package in R. Red: Gene expression is elevated. Green: Gene expression is reduced. Grey: Genes are expressed indifferent. (**a**) IgG4-ROD, (**b**) NSOI, (**c**) MALT lymphoma.

Observing the extracellular matrix (ECM)–receptor interaction compounds, IgG4-ROD samples had transcripts responsible for ECM adhesion altogether with their integrin domains downregulated compared to MALT lymphoma samples; namely, collagen, laminin, thrombospondin (THBS), α1, α 5, α 6, α 7 and β4 (ITGB4). Syndecan showed upregulation for both IgG4-ROD and MALT (Figure 5).

**Figure 6:**
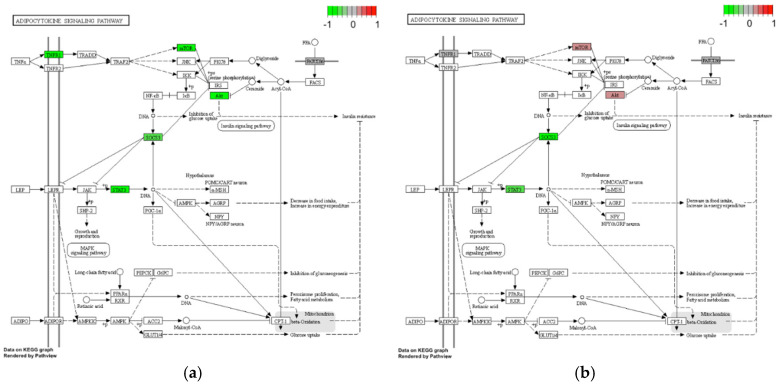

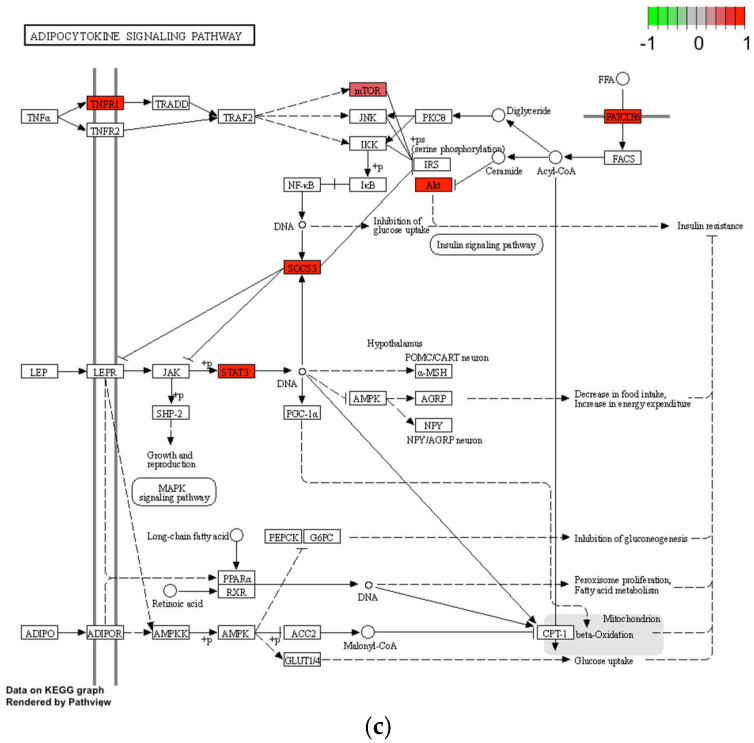
Differential gene expression of genes contributing to the adipocytokine signaling pathway interaction of different entities. The plots were created via the pathview package in R. Red: Gene expression is elevated. Green: Gene expression is reduced. Grey: Genes are expressed indifferent. (**a**) IgG4-ROD (**b**) NSOI. (**c**) MALT lymphoma.

Investigating the adipocytokine signaling pathway MALT lymphoma showed the strongest expression of *TNFR1*, *mTOR*, *AKT1* (*p* < 0.0079, FDR *p* < 0.0192), *SOCS3* (*p* < 0.009, FDR *p* < 0.019) and *STAT3* (*p* < 0.0025, FDR *p* < 0.0128), followed by NSOI showing a slight upregulation of mTOR and AKT compared to overall downregulation of compounds in IgG4-ROD (Figure 6).

## 4. Discussion

The present study provides the first signaling pathway analysis of IgG4-ROD, NSOI and orbital MALT lymphoma. We could show that there are specific differences in the activation and downregulation of signaling pathways between these entities.

Our previously published paper included differentially expressed genes mentioned in this paper. For a more distinctive approach, genes were implemented in a thorough machine learning algorithm. The main goal was to create a tool for diagnostic purposes, which was accomplished in identifying 35 (of 1364) biomarker genes. After receiving multiple questions about the possible origin and mechanisms resulting in these differentially expressed genes, we performed a gene set enrichment analysis for this study to provide a more detailed insight into the pathophysiology.

### 4.1. ECM–Receptor Interaction and Adipocytokine Signaling Pathway

Rosenbaum et al. recently published a study explaining IGF1-R and PPARγ enrichment in multiple OID (Graves’ orbitopathy, granulomatosis with polyangiitis, sarcoidosis and NSOI)), but not IgG4-ROD. Validation of our results is supported by the expression levels in the adipocytokine signaling pathway of NSOI when compared to the pathway analysis [17]. Mainly *mTOR, AKT, SOCS3 and STAT3* that also project insulin resistance effects through various downstream ligands. In contrast to the reduced expression in IgG4-ROD and NSOI samples, we see elevated expression levels in MALT samples. MALT samples showed diverging results when compared to all other OID, which was expected due to the nature of the disease from a biological point of view, though from a clinical approach the differentiation is not this easy [28]. A main focus of Rosenbaum et al. was the IGF-1R signaling pathway and downstream ligands (MAPK/RAS/RAF/MEK/ERK and PI3K/Akt/mTOR pathways). This is due to the recent FDA approval of an IGF1-R antibody (teprotumumab) for the treatment of GO. Due to the promising therapeutic effect of this first targeted therapy for GO it is of special interest to elucidate whether the agent can also be used for other OID [9]. Comparing healthy controls, Rosenbaum et al. found significantly altered gene expression within these pathways, which is why the authors concluded that GPA, sarcoidosis and NSOI patient might also profit from blockade of IGF-1R signaling pathways. Looking at our results regarding differences in IGF1 expression levels and activation of the IGF-1R downstream pathways (adipocytokine pathway), this hypothesis is supported. However, since we did not focus on these pathways, but had a broader approach, not all IGF-1R signaling and downstream pathways were included in our analysis.

Adipocytes themselves have been described as contributors to fibrosis many times before. Adipokines such leptin and adiponectin have been implicated to hepatic fibrosis [37], orbital fibrosis [38] and lung fibrosis [39]. Distinctive adhesion mechanisms are involved in epitheliotropic processes forming grounds of interaction between lymphoid and epithelial cells. The specific position of lymphoid cells within tissues results from numerous cell–cell and cell–matrix interactions that mediate the migration of lymphoid cells through extracellular matrix (ECM) receptors, from the bloodstream into the different compartments of the tissue [40]. Both IgG4-ROD and MALT lymphoma tissues showed significant activation of the ECM–receptor pathway, suggesting its important role in the orbital fibrosis in these entities. Rosenbaum et. al. already compared gene expression in tissue from patients with orbital fibrosis and pulmonary fibrosis [41]. They noted that transcripts characteristic such as fibronectin, lumican, thrombospondin, and collagen types I and VIII are common in both orbital and pulmonary fibrosis. Results from our study support this hypothesis. Comparison of compound expression in MALT lymphoma to IgG4-ROD probes (Figure 4 and Figure 5) indicates even stronger expression for transcript characteristic of ECM–receptor interaction in lymphoma than in IgG4-ROD. However, this observation could also be linked to the fact that orbital fat was more present in MALT lymphoma than in IgG4-ROD specimens. Integrins are transmembrane receptors that take part in the regulation of cell interactions with the extracellular matrix [42]. Integrins elevated in the lymphoma group through our study included: α1, α5, α6, α7 and β4 (ITGB4). ITGB4 binds exclusively with α6 and functions as a receptor for the membrane protein laminin. ITGB4 expression was already described in various malignant tumors including prostate cancer [43] and breast cancer [44]. Integrin-β4-targeted cancer immunotherapies have been described as possible therapeutics inhibiting growth and decreasing metastasis [45]. For ocular MALT lymphomas, this is the first study describing its altered expression. It should be further investigated as possible therapeutic target.

### 4.2. NOD-like Receptor Signaling Pathway

The innate immune system is the first line of defense against microbial invasion, relying on pattern recognition receptors to recognize external pathogenic microorganisms and then remove them [46]. The nucleotide-binding oligomerization domain (NOD) proteins, NOD1 and NOD2, represent two well-characterized pathogen recognition receptors (PRRs) [47,48]. The NOD-like receptor, belonging to the (NLR) family, detect conserved fragments found in the cell wall of many types of bacteria and activates intracellular signaling pathways. Detection is realized through intracellular sensors of pathogen-associated molecular patterns (PAMPs) that enter the cell via phagocytosis or pores, and damage-associated molecular patterns (DAMPs) that are associated with cell stress. NODs then drive proinflammatory and antimicrobial responses in different parts of the organism with TLRs, and interact with each other on different levels to regulate the immune response in the body. A recent case report by Harb. A. et al. [49] described a patient suffering acute vision loss from IgG4-related and bacterial rhinosinusitis after a COVID-19 infection, improving on corticosteroid and antibiotic treatment. This pathway being one of the prominent activated pathways in the IgG4-ROD group supports the hypothesis of microorganisms being a conductor in the activation of inflammation leading to a chronic disease that exacerbates in IgG4-immunopositive plasma cell infiltration [50]. Furthermore, a recent next-generation sequencing study of orbital MALT lymphoma in an Asian population revealed a higher occurrence of somatic mutations in the NOD-like receptor signaling pathway, suggesting its importance in the oncogenesis [51].

Information about expression levels of NOD-Like receptor signaling pathway in our MALT samples has been added as Supplementary Figure S1.

### 4.3. Outlook into B- and T-Cell Receptor Signaling

Interestingly both groups showed a decreased enrichment score for the B-cell receptor signaling pathway, T-cell receptor signaling pathway and Neurotrophin signaling pathway.

#### 4.3.1. B-Cell Receptor Signaling in MALT

Typically, a B-cell is defined and created by the productive rearrangement of immunoglobulin heavy (IgH) and light (IgL) chain genes, leading to expression of a B-Cell receptor (BCR). Each B-cell is encoded by a unique molecular fingerprint that results of unique sequences of it IgH and IgL hypervariable regions (HVRs) [52]. In gastric MALT lymphoma local self- and foreign antigens, provide direct antigenic stimulation of the tumor cells via their B-cell receptor. B-cells in MALT tend to express polyreactive, somatically mutated immunoglobulins after rearrangement in germinal centers, caused by the infectious agents [53]. These polyreactive (rheumatoid) autoantigens, that are responsible for Tumor progression function as an external activation mechanism for the NF-κB pathway [54]. This could explain the decrease in physiological BCR signaling for the MALT lymphoma Group in our study [24]. Chronic infection with *Chlamydia* spp. could be a co-factor in some cases for this progression as previous studies observed, similar to the common oncogenesis of gastric MALT lymphoma following chronic gastritis with Helicobacter pylori infection [55]. Others found no such infection [56,57,58] in ocular MALT lymphoma, demonstrating the inter-tumor heterogeneity in oncogenesis.

#### 4.3.2. B-Cell Receptor Signaling in IgG4-ROD

After recognition of IgG4-ROD as a unique disease entity, the humoral immune response received much attention, principally because of the hypergammaglobulinemia and the prominent IgG4-positive cells in both affected tissue and patient blood samples, disappearing upon corticosteroid treatment [59]. Further studies implicated the finding of autoreactive B-cell clones supporting the evidence that humoral immune response in IgG4-RD was directed towards self-antigens [60,61]. A reduction of circulating plasmablast numbers in patients with IgG4-RD following remission induced by B-cell depletion has also been reported [62]. Clinically visual compelling data for pathogenic role of B-Cell involvement, is the therapeutic effect of B-cell depletion with rituximab therapy on IgG4-ROD [63,64]. Although corticosteroids were tempered before surgery, IgG4-ROD samples received corticosteroid therapy weeks before acquisition, possibly explaining the reduced BCR signaling in our study. However, the humoral immune response alone is not sufficient to explain the pathophysiology of IgG4-ROD.

#### 4.3.3. T-Cell Receptor Signaling and Cytokine Induced Proliferation

Previously mentioned B-cell expansion taking place in secondary lymphatic tissue via iterative rounds of mutation and positive selection within germinal centers, is a T-cell-dependent process. In vitro studies have shown that T follicular helper cells (Tfh) associated with different Th subsets differentially shape the quality of human humoral immunity. A study investigated the role of CXCR5+ CD4+ T-cells circulating through the human blood [65]. Their results, in accordance with other studies [66,67,68], showed that induced naive and memory B-cells become Ig-producing cells via IL-21, IL-10 and ICOS, and secreted CXCL13. T-cells in this study were composed of three different subsets: Th1, Th2 and Th17. CXCR5+ Th2 and CXCR5+ Th17 cells induced naïve B-cells to secrete Igs through IL-21. However, CXCR5+ Th2 modulated the isotype switch differently to CXCR5+ th17 cells. CXCR5+ Th2 cells promoted IgG and IgE secretion in contrast to CXCR5+ Th17 cells promoting IgG and IgA secretion. In our study, Th17 cell differentiation was significantly reduced in MALT group, pointing to a possibly similar Th-12/Th-17 induced differentiation in observed samples (Figure 2). Furthermore, T follicular regulator cells (Tfr) have been also proposed as a regulator in T-cell cytokine induced B-cell proliferation [69]. In IgG4-ROD, it is possible that the relative amount of IL-4-producing and IL-10-producing T-cells determines whether a given B-cell class switches to IgG4 or Ig [70,71]. Our study showing the cytokine–cytokine receptor interaction pathway being enriched in IgG4-ROD (Figure 3) supports this hypothesis. In addition, our study showed a decrease in the physiological T-cell receptor signaling pathway. In accordance with the previously mentioned studies, we hypothesize a relative imbalance of both T helper cells and cytokines causing an autoreactivity in early B-cell development leading to impaired regulation and activation of peripheral B-cells by different forms of self or foreign antigens and T-cell crosstalk. This could lead to a cytokine induced fibroblast recruitment on a derived ECM–receptor interaction causing fibrosis through adipocytokine signaling and finally organ enlargement in form of tumor-like soft tissue mass. The progression is mediated through various pathways in which endogen and exogen activation of IGF-1R signaling, NOD-like receptor signaling, B- and T-cell receptor signaling could play a key role.

### 4.4. Limitations

Limitation of this study include its relatively small number of patients that are collected from only one institute and its retrospective design. This might have resulted in selection and confounding bias. Furthermore, the heterogeneous histopathological findings and orbital locations might mask gene expression differences that are only present in specific subtypes of the entities, especially in NSOI. However, since the main goal of this study was to differentiate pathophysiological aspects between NSOI, IgG4 and MALT, this broader analysis revealed the most prominent pathways for each entity. Further analysis of IgG4-ROD and GO should be conducted via elementary methods, such as immunoassays and in cell cultures, before serious therapeutic assumptions can be drawn. We see a validation of our results comparing IgG4-ROD and NSOI pathway analysis with other study groups. A previous analysis of OID with neoplastic disorders in this form has only been conducted in our previously published study, which makes a literature review for validation purposes difficult. However, looking at compound expression of our preformed GSEA, we can see a validation of molecular mechanisms as expected.

## 5. Conclusions

In our study, we show that though NSOI, IgG4-ROD and orbital MALT lymphoma share clinical symptoms, the enriched pathways are significantly different. Especially, MALT lymphoma showed completely different pathway patterns, i.e., elevated ECM–receptor interaction and adipocytokine pathways in contrast to downregulations in IgG4-ROD and NSOI samples. In contrast, NSOI was prominent in the activation of innate immune pathways; both IgG4-ROD and MALT showed significantly decreased enrichment in definitive cell differentiation conductors: B- and T-cell signaling pathways. These common pathways support the hypothesis that chronic inflammation of IgG4-ROD could lead to orbital MALT lymphoma by malignant transformation of lymphocytes.

## Figures and Tables

**Table 1:** Differentially expressed count analysis in MALT lymphoma (compared to IgG4-ROD and NSOI) for *IGF-1* (*p* < 0.01, FDR *p* < 0.02), IGFBP4 (*p* < 0.001, FDR *p* < 0.0122) and IGFBP7 (*p* < 0.005, FDR *p* < 0.02).

Gene	*p*-Value	FDR-Adjusted *p* Values	Lower CI	Higher CI
Lymphoma by *IGF1*	0.0110	0.0224	−349.7	−59.2
Lymphoma by *IGFBP4*	0.0008	0.0121	−2204.0	−783.1
Lymphoma by *IGFBP7*	0.0052	0.0165	−1670	−407.3

## Data Availability

The data presented in this study are available on request from the corresponding author. The data are not publicly available due to local data regulations.

## Supplementary Materials

The following are available online at https://www.mdpi.com/article/10.3390/life12101660/s1. Figure S1: Differential gene expression of genes contributing to the NOD like receptor signaling pathway in MALT samples. The plots were created via the pathview package in R. Red: Gene expression is elevated. Green: Gene expression is reduced. Grey: Genes are expressed indifferent, Table S1: (a) nCounter^®^ Human Immunology V2 Panel—Gene and Probe Details. (b) nCounter^®^ PanCancer Progression Panel—Gene and Probe Details.
