# Supplementary material for: Novel Insights into Pathophysiology of Orbital Inflammatory Diseases and Progression to Orbital Lymphoma by Pathway Enrichment Analysis
Source: Life (Basel). 2022 Oct 20;12(10):1660. doi: 10.3390/life12101660 (PMC9605068; doi:10.3390/life12101660)
Supplement: Supplementary file 1 [file Supplementary Figure S1.pdf › Supplementary Figure S1.pdf]

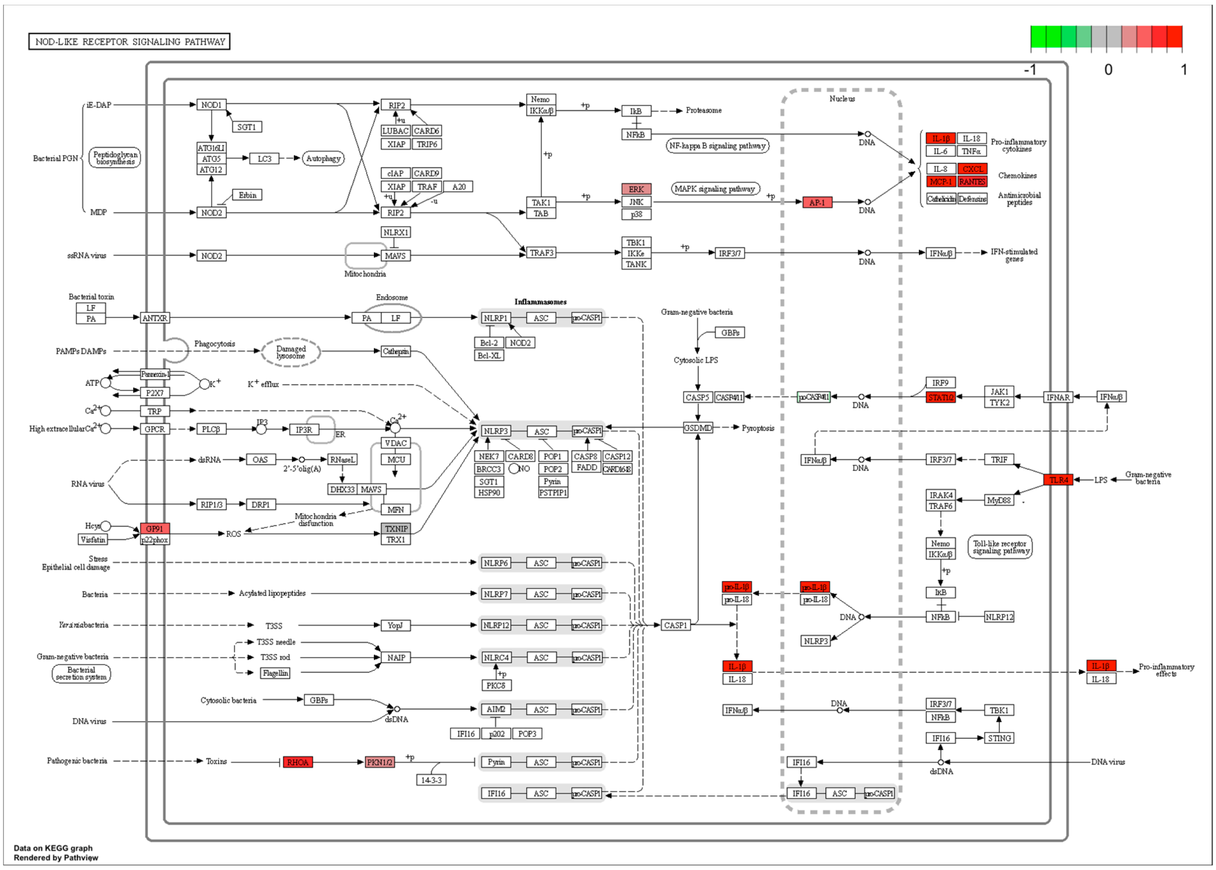

Figure S1. Differential gene expression of genes contributing to the NOD like receptor signaling pathway in MALT samples. The plots were created via the pathview package in R. Red: Gene expression is elevated. Green: Gene expression is reduced. Grey: Genes are expressed indifferent.
